# APOE4 alters vascular cells in cognitively normal brains and in vitro models revealing early pathogenic AD contributions of endothelial cells

**DOI:** 10.1002/alz70855_098686

**Published:** 2025-12-23

**Authors:** Taylor Bertucci

**Affiliations:** ^1^ Neural Stem Cell Institute, Albany, NY, USA

## Abstract

**Background:**

Breakdown of the blood brain barrier (BBB) occurs early in Alzheimer's Disease (AD) and AD‐related dementias (ADRD). APOE4 is a risk factor for vascular complications associated with AD, vascular dementia, and Cerebral Amyloid Angiopathy (CAA). Moreover, newly approved anti‐amyloid immune‐therapies are more likely to cause brain hemorrhage and edema in APOE4 carriers. Recent studies have demonstrated that cognitively normal aging APOE4 carriers have greater vascular leakage compared to non‐carriers and that this is exacerbated as AD develops. These observations implicate APOE4 in BBB impairment, but the underlying mechanisms remain unclear.

**Method:**

Combining several single nuclear RNAseq brain datasets enabled us to define gene expression differences in APOE4 carriers in endothelial cells (ECs), pericytes, smooth muscle cells, and fibroblasts in postmortem human brains. Using a mesoderm patterning process, we then generated pluripotent stem cell (iPSC)‐derived ECs and mural cells from 5 pairs of APOE4/4 and APOE3/3 isogenic lines. We used 3D self‐assembling vascular models to reveal vascular phenotypes across ECs, pericytes, smooth muscle cells and fibroblasts characterized by single‐cell RNAseq. We captured 2D EC monolayer barrier profiles measured by electric cell‐substrate impedance sensing (ECIS) to establish the functional impact of APOE genotype and to test drugs to improve EC barrier recovery after inflammation challenges.

**Results:**

In cognitively normal (control) brains, APOE4 ECs were enriched for interferon‐gamma pathway‐related genes and reduced oxidative phosphorylation. Several interferon‐gamma and oxidative phosphorylation pathway gene expression changes were also captured in the iPSC‐derived vascular models. 3D APOE4/4 vascular models showed increased fibronectin (FN1) deposition surrounding vessel structures, consistent with previous reports of increased FN1 deposition around blood vessels in the brains of APOE4 carriers with AD. This is notable as loss of function variants in FN1 can protect against the increased risk of AD due to APOE4. In 2D iPSC‐EC monolayers, we established functional differences in barrier strength and response to inflammation in APOE4/4 ECs, including reduced barrier recovery after a TNFα + IL1β inflammatory challenge.

**Conclusions:**

APOE4 genotype has an impact on vascular cells. Further analysis is ongoing to test drugs to recovery APOE4 barrier functionality and test vascular impact on neuronal health in neurovascular 3D models.

